# Incidence of ROTEM^®^-defined coagulopathy at hospital arrival among polytrauma patients receiving fibrinogen concentrate in prehospital setting: a prospective observational study

**DOI:** 10.1007/s00068-026-03183-8

**Published:** 2026-04-24

**Authors:** Kamil Vrbica, Filip Keller, Jan Stingl, Katerina Vanickova, Radka Stepanova, Martin Dolecek, Ondrej Hrdy, Roman Gal

**Affiliations:** 1 Department of Anaesthesiology and Intensive Care Medicine, University Hospital Brno, Brno, Czech Republic; 2 Department of Anaesthesiology and Intensive Care Medicine, Faculty of Medicine, Masaryk University, Brno, Czech Republic; 3 Faculty of Medicine, Masaryk University, Brno, Czech Republic; 4 Department of Pharmacology/CZECRIN, Masaryk University Faculty of Medicine, Brno, Czech Republic

**Keywords:** Fibrinogen, Blood coagulation tests, Trauma, Platelets, Point of care testing

## Abstract

**Purpose:**

Trauma-induced coagulopathy affects approximately one-quarter of polytrauma patients and is associated with increased mortality. Given the central role of fibrinogen and its early depletion after injury, supplementation is recommended after hospital admission in the presence of hypofibrinogenaemia or suspected major haemorrhage. As coagulopathy develops within minutes of trauma, prehospital fibrinogen administration may help mitigate its progression before hospital arrival. This study evaluated the incidence of coagulopathy at hospital admission, assessed using ROTEM^®^, among polytrauma patients receiving fibrinogen concentrate in the prehospital setting.

**Methods:**

In this single-centre, prospective observational study, adult patients with polytrauma (Injury Severity Score ≥ 16) were enrolled after prehospital administration of fibrinogen concentrate. The primary outcome was the presence of coagulopathy at hospital arrival, defined according to predefined ROTEM^®^ Delta thresholds (FIBTEM MCF ≤ 8 mm and/or EXTEM CT ≥ 80 s and/or EXTEM MCF ≤ 49 mm). The secondary outcomes were laboratory coagulation status, 28-day all-cause mortality, and thromboembolic complications.

**Results:**

Thirty patients were enrolled, of whom 28 were included in the final analysis. Twenty-seven patients received 4 g of fibrinogen concentrate and one received 2 g due to short transport time. ROTEM^®^-defined coagulopathy was identified in two patients (7.1%), both based on EXTEM abnormalities; no patient had reduced FIBTEM MCF. Five patients (17.9%) died within 28 days and two patients (7.1%) experienced thromboembolic complications.

**Conclusions:**

Our findings describe a low incidence of ROTEM^®^-defined coagulopathy at hospital arrival in polytrauma patients who received prehospital fibrinogen concentrate. Adequately powered randomized controlled trials are required to determine whether prehospital fibrinogen administration reduces coagulopathy and improves clinically relevant outcomes.

**Trial registration:**

NCT03572309, ClinicalTrials.gov, registered on 19 June 2018.

**Supplementary Information:**

The online version contains supplementary material available at 10.1007/s00068-026-03183-8.

## Background

Trauma-induced coagulopathy (TIC) is a complex dysregulation of haemostasis occurring in patients with severe traumatic injury [1, 2]. TIC is identified in approximately one in four severely injured patients after admission to the emergency department (ED) [3]. The presence of TIC is associated with a mortality more than threefold that observed in patients with trauma without coagulopathy [4]. The severity of coagulopathy can be objectively assessed with conventional coagulation tests, or with viscoelastic methods such as ROTEM^®^, TEG 6s^®^, or ClotPro^®^; the results can be subsequently used for goal-directed treatment [5]. The complex mechanism underlying TIC involves tissue injury, shock, endothelial dysfunction, and dysregulation of both the immune and coagulation systems. A key consequence is hypofibrinogenemia, driven primarily by fibrinogen consumption and hyperfibrinolysis [2]. Fibrinogen is typically the first coagulation factor to reach a critically low level during major haemorrhage, and its depletion is closely associated with increased transfusion requirements and mortality; consequently, fibrinogen supplementation is a crucial component of TIC management [6, 7]. Initial administration of fibrinogen according to clinical criteria indicating major haemorrhage and TIC risk is recommended in the European guidelines for management of major bleeding [8]. The fibrinogen is typically administered as fibrinogen concentrate or cryoprecipitate early after admission to the ED. However, the recent CRYOSTAT-2 trial did not demonstrate a mortality benefit from early empirical cryoprecipitate administration in polytrauma patients [9]. Nevertheless, given the rapid development of hypofibrinogenemia and hyperfibrinolysis after trauma, the question remains whether even earlier administration of fibrinogen at the scene might influence the subsequent development of coagulopathy [10, 11]. The aim of this study was to describe coagulation status at hospital arrival in polytrauma patients following prehospital administration of fibrinogen concentrate, as measured using the ROTEM^®^ Delta device.

## Methods

This single-centre prospective observational study was conducted at the Department of Anaesthesiology and Intensive Care Medicine, University Hospital Brno, between June 2018 and May 2023. This study adhered to the principles of Good Clinical Practice and the Declaration of Helsinki. Ethical approval was granted by the Ethics Committee of University Hospital Brno (approval No. 01-160518). The study was registered on 19 June 2018 on ClinicalTrials.gov (NCT03572309). Written informed consent was obtained from all enrolled patients. If patients had impaired capacity to provide consent, written informed consent was obtained from a legally authorized representative. If no legal representative was available at the time of enrolment, consent was obtained from an independent physician who was not involved in the study but was familiar with the study protocol.

The primary outcome was the incidence of coagulopathy at hospital arrival in polytrauma patients who had received fibrinogen concentrate during the prehospital phase. Coagulopathy was defined according to previously published criteria from the Philadelphia consensus panel commonly used to identify TIC, as a FIBTEM MCF ≤ 8 mm and/or EXTEM CT ≥ 80 s and/or EXTEM MCF ≤ 49 mm, measured using the ROTEM^®^ Delta device [12]. Secondary outcomes included conventional laboratory coagulation parameters at hospital arrival, 28-day all-cause mortality, and the occurrence of thromboembolic complications within 28 days after injury. Thromboembolic complications were defined as deep vein thrombosis or pulmonary embolism, confirmed by imaging.

Patients 18 years or older who presented to the ED with polytrauma (defined as an Injury Severity Score (ISS) ≥ 16) and who received fibrinogen concentrate during the prehospital treatment phase were included in the study. Fibrinogen administration followed local emergency medical service protocols based on vital signs and suspected bleeding-related injuries, as described in Supplement 1. Patients with ISS = 75, pregnant patients, and patients receiving therapeutic doses of anticoagulant and/or antiplatelet drugs were excluded from the study.

Patient data were collected through paper case report forms. Demographic data and vital functions from the site of injury (including systolic and diastolic blood pressure, heart rate, and Glasgow coma scale score) were extracted from emergency medical service records. The same vital functions were recorded again immediately after ED arrival. Simultaneously, core body temperature was recorded, and blood samples for a standardized set of blood tests (S-Monovette^®^ EDTA K3E/2.7 ml; Sarstedt AG, Nümbrecht, Germany; S-Monovette^®^ Citrate 9NC/3.0 ml; Sarstedt AG, Nümbrecht, Germany) and standardized viscoelastic method tests (S-Monovette^®^ Citrate 9NC/3.0 ml; Sarstedt AG, Nümbrecht, Germany) were obtained. The laboratory tests included haemoglobin level, platelet count, prothrombin time, international normalized ratio, activated partial thromboplastin time, thrombin time, fibrinogen level, lactate level, and base excess.

The viscoelastic method tests were conducted with a ROTEM^®^ Delta device (Tem Innovations GmbH, Munich, Germany) and included EXTEM S and FIBTEM S assays. All measurements were performed in point-of-care testing mode by a trained attending physician, according to the manufacturer’s instructions. The recorded viscoelastic method parameters for both assays were clotting time (CT), alpha angle (α), clot formation time (CFT), clot amplitude at 10 min (A10), maximum clot firmness (MCF) and maximum lysis (ML). A detailed description of the tests used is provided in Supplement 2. The ISS was calculated later after admission. After 28 days, the mortality outcome and potential thromboembolic complications were assessed. The study is reported in accordance with the Strengthening the Reporting of Observational Studies in Epidemiology (STROBE) guidelines [13].

No formal sample size calculation for clinical outcomes was performed, and the study was not powered to evaluate clinical outcomes. All parameters collected in this observational study were analysed descriptively. Categorical variables are presented as absolute and relative frequencies, whereas continuous variables are summarized with mean and standard deviation, median with first and third quartiles, and minimum and maximum. Statistical analysis was conducted in SAS version 9.4.

## Results

Thirty patients were initially enrolled in the study. After review of the complete clinical data, two patients were found to meet the exclusion criteria and were therefore excluded: one due to therapeutic-dose anticoagulation and the other due to an ISS of 75. Therefore, twenty-eight patients were included in the final analysis. The median (IQR) GCS at the scene was 10 (4–14), the median systolic blood pressure was 111 mmHg (82–133), and the median diastolic blood pressure was 67 mmHg (50–89), the median shock index was 1.0 (0.7–1.3) calculated at 26 patients, two patients in cardiac arrest were excluded due to absent measurable systolic blood pressure. A fibrinogen concentrate dose of 4 g was administered to 27 patients (96.4%), whereas one patient (3.6%) received 2 g because of a short transportation time to the hospital. Patient characteristics at admission to the ED are summarized in Table 1.

**Table 1:** Patients’ characteristics upon admission to the Emergency department

Parameter			Patients (*N* = 28)
Gender, n (%)		Female	4 (14.3)
		Male	24 (85.7)
Age, mean (± SD), years			45 (± 15.6)
BMI, median (IQR), kg/m^2^			26.1 (24.0-27.8)
Type of injury, n (%)		Blunt	28 (100)
		Penetrating	0 (0)
TBI, n (%)		No	15 (53.6)
		Yes	13 (46.4)
ISS, median (IQR)			30 (21–39)
Invasive mechanical ventilation, n (%)		No	5 (17.9)
		Yes	23 (82.1)
Tranexamic acid in prehospital care, n (%)		No	0 (0)
		Yes	28 (100)
Noradrenaline, mean (± SD), µg/kg/min			0.02 (± 0.042)
Body temperature, median (IQR), °C			36.3 (35.7–36.6)
Heart rate, median (IQR), bpm			102 (87–120)
Systolic blood pressure, median (IQR), mmHg			112 (85–135)
Diastolic blood pressure, median (IQR), mmHg			80 (53–100)
Shock index, median (IQR)			1.0 (0.6–1.5)

SD – Standard deviation; BMI – Body Mass Index; TBI – Traumatic Brain Injury; ISS – Injury Severity Score; IQR, interquartile range

Laboratory parameters and ROTEM^®^ findings after ED admission are presented in Tables 2 and 3.

**Table 2:** Laboratory parameters upon admission to the Emergency department

Parameter	Patients (*N* = 28)
Haemoglobin, median (IQR), g/L	123 (110–134)
Platelets, median (IQR) [10^9/L]	225 (188–274)
INR, median (IQR)	1.04 (0.97–1.19)
Prothrombin time, median (IQR), s	15.1 (13.7–16.3)
Thrombin time, median (IQR), s	24.1 (20.6–25.2)
aPTT, median (IQR), s	32.3 (28.5–37.0)
Fibrinogen, median (IQR), g/L	3.23 (2.87–3.39)
Base excess, median (IQR), mmol/L	-2.8 (-6.4-0.8)
Lactate, median (IQR), mmol/L	2.8 (1.6–3.6)

INR, International normalised ratio; aPTT, Activated partial thromboplastin time; IQR, interquartile range

**Table 3:** EXTEM and FIBTEM parameter characteristics

Parameter	Statistics	EXTEM (*N* = 28)	FIBTEM (*N* = 28)
CT [s]	n / missing	28 / 0	28 / 0
	Median (IQR)	56 (50–62)	54 (47–58)
	Min-Max	25–80	16–82
CFT [s]	n / missing	28 / 0	9 / 19
	Median (IQR)	122 (99–147)	1624 (825–2537)
	Min-Max	71–290	227–3255
α [°]	n / missing	28 / 0	27 / 1
	Median (IQR)	74 (67–79)	75 (64–79)
	Min-Max	58–83	44–82
A10 [mm]	n / missing	28 / 0	28 / 0
	Median (IQR)	52 (47–56)	13 (11–15)
	Min-Max	30–62	8–25
A20 [mm]	n / missing	28 / 0	28 / 0
	Median (IQR)	58 (56–63)	14 (11–18)
	Min-Max	37–69	9–23
MCF [mm]	n / missing	28 / 0	28 / 0
	Median (IQR)	60 (58–64)	14 (11–19)
	Min-Max	38 / 69	9 / 25
ML [%]	n / missing	23 / 5	23 / 5
	Median (IQR)	6 (3–8)	5 (0–13)
	Min-Max	1–15	0–64

α, alpha angle; CT, clotting time; CFT, clot formation time; A5, amplitude at 5 min; A10, amplitude at 10 min; A20, amplitude at 20 min; MCF, maximum clot firmness; ML, maximum lysis; IQR, interquartile range

Five patients (17.9%) died by day 28, and two patients (7.1%) experienced thromboembolic complications. Coagulopathy, as defined by ROTEM^®^ measurements, was observed in two patients (7.1%): one with an EXTEM CT ≥ 80 s and the other with an EXTEM MCF ≤ 49 mm. No patients met the criterion of FIBTEM MCF ≤ 8 mm. No patients with coagulopathy died within the 28-day follow-up period.

## Discussion

To our knowledge, this study is only the second to report the effects of prehospital fibrinogen administration on coagulation status, viscoelastic parameters, and the incidence of ROTEM^®^-defined coagulopathy corresponding to TIC definition in patients with polytrauma. Among 28 patients with polytrauma, residual coagulopathy after tranexamic acid and fibrinogen concentrate administration was identified in only two patients using viscoelastic testing. This incidence observed in our cohort is substantially lower than that reported in previous cohorts of similarly injured patients, however, given the small sample size, no definitive conclusions can be drawn regarding a potential protective effect of early fibrinogen supplementation on the development or persistence of TIC [4, 14].

To date, the only published study specifically addressing prehospital fibrinogen administration, by Ziegler et al. [15], has demonstrated that prehospital administration of fibrinogen concentrate is feasible and significantly improves clot stability. However, that study did not report coagulopathy incidence after ED admission or mortality outcomes. Although both our study and that by Ziegler et al. included 28 patients treated with prehospital fibrinogen, and had comparable distributions of age, sex, BMI, and traumatic brain injury rate, our cohort exhibited a higher median ISS (30 vs. 25), a lower initial Glasgow Coma Scale score (10 vs. 14.5), and a greater proportion of patients intubated at the scene (23 vs. 11). These differences indicate that our cohort was more severely injured. Nonetheless, owing to the absence of outcome data in the study by Ziegler et al., the ability to make direct comparisons remains limited. However, the incidence of coagulopathy corresponding to TIC definition after hospital admission in our cohort can be compared with previously published cohorts of trauma patients who did not receive prehospital transfusion products or blood derivatives in prehospital care. Unfortunately, the available data remain limited, and potential comparisons are constrained by heterogeneity in patient populations, injury severity, and variations in the definition of TIC. Overall, the incidence of TIC has been reported to be approximately 25% [3], in agreement with the findings of Tauber et al., who observed rates of 22–30%, depending on the ROTEM^®^ parameter assessed, and with those of Asim et al., who reported abnormal ROTEM^®^ results in 25.3% of severely injured patients [16, 17]. In a study by Innerhofer et al., 100 of 254 assessed patients met the ROTEM^®^ criteria for treatment [18], which were nearly identical to those in our definition of ROTEM^®^-defined coagulopathy. This finding corresponds to an incidence of approximately 40%, In contrast, a recent study by Richards et al. reported fibrinogen levels below 1.5 g/L in only 4.8% of polytrauma patients upon arrival at the ED [19]. However, this analysis was limited to Clauss-measured fibrinogen concentrations and did not assess the broader haemostatic profile by viscoelastic methods.

Other studies have investigated fibrinogen administration in the early in-hospital management of traumatic bleeding. Fibrinogen was typically administered either as fibrinogen concentrate or cryoprecipitate. Both forms of fibrinogen supplementation appear to have similar effectiveness; however, the time required for administration with fibrinogen concentrate is approximately half that with cryoprecipitate, thus enabling more rapid TIC reversal [20]. A comparison of patients with trauma treated with fibrinogen concentrate vs. cryoprecipitate has indicated that those receiving fibrinogen concentrate have lower requirements for red blood cells, fresh frozen plasma, and platelet transfusions, and have shorter lengths of hospital and intensive care unit stays, as illustrated in a retrospective cohort study by Obadi et al. [21]. Itagaki et al. have conducted a propensity-matched cohort study comparing patients with trauma who received fibrinogen concentrate within 1 h after ED arrival vs. within 1–24 h after admission. Despite receiving comparable total amounts of fibrinogen concentrate, the group treated within 1 h after arrival had a significantly lower in-hospital mortality rate (19.3% vs. 45%, *p* = 0.03) [22]. This finding suggests that the earliest possible administration of fibrinogen, including in the prehospital setting, might yield a similar effect. In contrast, the recent large, randomized CRYOSTAT-2 trial, in which empiric high-dose cryoprecipitate was administered to bleeding trauma patients within a major haemorrhage protocol, did not demonstrate a reduction in 28-day mortality or improvement in secondary clinical outcomes. In that study, the median time from injury to hospital arrival was 75 min and the median time from admission to cryoprecipitate administration was 68 min, resulting in a median time of approximately 2.5 h from injury to fibrinogen supplementation. In the subgroup of patients with penetrating trauma, mortality was higher in the cryoprecipitate group, whereas in patients with blunt trauma, mortality was numerically lower compared with standard care. Although these subgroup findings should be interpreted cautiously, they suggest possible heterogeneity of treatment effect. The underlying mechanisms remain unclear and warrant further investigation. Factors such as the time required for fibrinogen administration, as well as the mechanism of injury, may have influenced outcomes. These observations raise the question of whether both earlier fibrinogen administration and patient selection based on injury characteristics could yield different clinical effects. Additionally the recent randomized FiiRST-2 trial did not demonstrate superiority of clotting factor concentrates over fresh frozen plasma for initial resuscitation in polytrauma patients requiring activation of a massive bleeding protocol in the in-hospital setting [23]. While retrospective studies have reported associations between early fibrinogen supplementation and improved outcomes, randomized controlled trials have not demonstrated a clinical benefit.

Our study demonstrated a 28-day mortality rate of 17.9%, which is similar to those reported in the fibrinogen groups in a recent meta-analysis by Burt et al. evaluating fibrinogen concentrate administration within 4 h after hospital admission (18.0%) [18, 24–26]. Our cohort had a 7.1% incidence of thromboembolic complications, which is similar to the 9% reported in the fibrinogen concentrate group by Obaid et al. and the 8% reported in the fibrinogen concentrate group by Innerhofer et al. [18, 21]. Both studies aimed to rapidly administer fibrinogen concentrate to severely injured patients with trauma with TIC at hospital admission. However, given the single-arm design and the limited sample size with a low number of events, these observations should be interpreted with caution and cannot be considered clinically conclusive.

On the basis of current evidence, the clinical benefit of early fibrinogen administration remains uncertain. While observational data have indicated possible benefits, large randomized trials have not demonstrated similar effects. With regard to the current evidence on prehospital fibrinogen concentrate administration, Ziegler et al. demonstrated that prehospital fibrinogen administration is feasible and increases clot stability [15]. However, its impact on clinically relevant outcomes has not been established. In our study, the rates of mortality and the incidence of thromboembolic complications were within the range reported in previously published studies evaluating patients who received fibrinogen concentrate after hospital admission [18, 21, 24–26]. However, this represents an indirect comparison across studies, and given the small sample size of our study, no conclusions regarding comparative effectiveness or safety can be drawn. Compared with previously reported trauma cohorts, our patients demonstrated approximately one-quarter the incidence of ROTEM^®^-defined coagulopathy corresponding to TIC definition at hospital arrival [16–18]. TIC is an independent predictor of adverse outcomes and mortality, earlier correction of coagulopathy remains biologically plausible as a therapeutic target. Although observational and retrospective studies have suggested potential benefits of early in-hospital fibrinogen administration, randomized controlled trials have not demonstrated reductions in mortality or other clinically relevant outcomes. Accordingly, the overall clinical impact of prehospital fibrinogen supplementation remains to be established, and large, adequately powered randomized trials are required.

Our study has several limitations, including the use of a non-clinical parameter as the primary endpoint. The use of a non-clinical endpoint was necessitated by anticipated limited patient recruitment, further affected by the COVID-19 pandemic. Nevertheless, the sample size is comparable to that of the only similar published study [15]. Given that clinical outcomes, including mortality and thromboembolic complications, were assessed only as secondary endpoints, and considering the heterogeneity of the trauma population and the limited sample size, no definitive conclusions can be drawn regarding the effects of prehospital fibrinogen administration on these outcomes. Another limitation is the study’s observational design, including the absence of a control group for direct comparison and the lack of baseline coagulation assessment prior to prehospital fibrinogen administration. Additionally, the decision to administer fibrinogen was made independently by the emergency medical service team according to a local protocol, which was not based on established prediction tools such as the Trauma associated severe haemorrhage score or the Traumatic bleeding severity score [27, 28]. The main strength of this study is the precise description of haemostasis and the incidence of coagulopathy, assessed with ROTEM^®^, in a well-defined cohort of polytrauma patients who received fibrinogen concentrate in the prehospital setting.

## Conclusions

Our findings describe a low incidence of ROTEM^®^-defined coagulopathy consistent with established definitions of TIC at hospital arrival in polytrauma patients who received prehospital fibrinogen concentrate. Large randomized controlled trials are required to establish whether prehospital fibrinogen administration reduces the incidence of coagulopathy and to clarify its potential impact on transfusion requirements, morbidity, and mortality in trauma patients.

## Supplementary Information

Below is the link to the electronic supplementary material.

Supplementary Material 1

Supplementary Material 2

## Data Availability

The datasets used and/or analysed during the current study are available from the corresponding author on reasonable request.

## Abbreviations

α: alpha angle
A10: Clot firmness at 10 minutes
aPTT: Activated partial thromboplastin time
CFT: Clot formation time
CT: Clotting time
ED: Emergency department
GCS: Glasgow coma scale
INR: International normalised ratio
IQR: Interquartile range
ISS: Injury severity score
MCF: Maximum clot firmness
ML: Maximum lysis
SD: Standard deviation
TIC: Trauma–induced coagulopathy
