# Supplementary material for: Incidence of ROTEM®-defined coagulopathy at hospital arrival among polytrauma patients receiving fibrinogen concentrate in prehospital setting: a prospective observational study
Source: Eur J Trauma Emerg Surg. 2026 Apr 24;52(1):144. doi: 10.1007/s00068-026-03183-8 (PMC13109142; doi:10.1007/s00068-026-03183-8)
Supplement: Supplementary file 1 — Supplementary Material 1 [file 68_2026_3183_MOESM1_ESM.docx]

# Supplement 1

# Fibrinogen indication criteria

## Vital Signs

| **Parameter** | **Measurement** | **Points** |
| --- | --- | --- |
| Systolic blood pressure  or capillary refill time | < 90 mmHg  > 4 s | 5  5 |
| Heart rate | > 100 bpm | 1 |

## Suspected Source of Bleeding

| **Location** | **Clinical finding** | **Points** |
| --- | --- | --- |
| Chest | Chest instability | 1 |
| Abdomen | Peritoneal irritation | 1 |
| Pelvis | Pelvic instability | 2 |
| Left lower limb | Long bone fracture | 1 |
| Right lower limb | Long bone fracture | 1 |

## Treatment Recommendation

Fibrinogen concentrate administration: 4 g (2 vials) if score ≥ 7
