# Supplementary material for: Incidence of ROTEM®-defined coagulopathy at hospital arrival among polytrauma patients receiving fibrinogen concentrate in prehospital setting: a prospective observational study
Source: Eur J Trauma Emerg Surg. 2026 Apr 24;52(1):144. doi: 10.1007/s00068-026-03183-8 (PMC13109142; doi:10.1007/s00068-026-03183-8)
Supplement: Supplementary file 2 — Supplementary Material 2 [file 68_2026_3183_MOESM2_ESM.docx]

**Supplement 2**

**ROTEM® Delta device performed tests**

- EXTEM S (EXTEM, activated extrinsic pathway with tissue factor)
- FIBTEM S (FIBTEM, activated extrinsic pathway with tissue factor in the presence of the platelet inhibitor cytochalasin D)
- clotting time (CT; the time from coagulation activation to a 2 mm clot firmness, measured in seconds)
- alpha angle (α, measured in degrees)
- clot formation time (CFT; the time from 2 to 20 mm clot firmness, measured in seconds)
- clot amplitude 10 minutes after CT clot formation time (A10, measured in mm)
- maximum clot firmness (MCF; the maximum signal amplitude reflecting clot strength, measured in mm)
- maximum lysis (ML; the percentage of MCF decrease during measurement)
